# Evaluating the Performance and Safety of Large Language Models in Generating Type 2 Diabetes Mellitus Management Plans: A Comparative Study With Physicians Using Real Patient Records

**DOI:** 10.7759/cureus.80737

**Published:** 2025-03-17

**Authors:** Agnibho Mondal, Arindam Naskar, Bhaskar Roy Choudhury, Sambudhya Chakraborty, Tanmay Biswas, Sumanta Sinha, Sasmit Roy

**Affiliations:** 1 Department of Infectious Diseases and Advanced Microbiology, School of Tropical Medicine, Kolkata, IND; 2 Department of Endocrinology, Nutrition and Metabolic Diseases, School of Tropical Medicine, Kolkata, IND; 3 Department of Geriatric Medicine, Medical College and Hospital, Kolkata, Kolkata, IND; 4 Department of Infectious Diseases, All India Institute of Medical Sciences, New Delhi, New Delhi, IND; 5 Department of Internal Medicine, Debra Super Speciality Hospital, Kolkata, IND; 6 Department of Medicine and Critical Care, Shuvadarsini Multi-Speciality Hospital, Durgapur, IND; 7 Department of Nephrology, University of Virginia, Lynchburg, USA

**Keywords:** artificial intelligence in medicine, clinical decision support system, diabetes mellitus type 2, large language model(llm), oral hypoglycemic agents

## Abstract

Background

The integration of large language models (LLMs) such as GPT-4 into healthcare presents potential benefits and challenges. While LLMs show promise in applications ranging from scientific writing to personalized medicine, their practical utility and safety in clinical settings remain under scrutiny. Concerns about accuracy, ethical considerations, and bias necessitate rigorous evaluation of these technologies against established medical standards.

Methods

This study involved a comparative analysis using anonymized patient records from a healthcare setting in the state of West Bengal, India. Management plans for 50 patients with type 2 diabetes mellitus were generated by GPT-4 and three physicians, who were blinded to each other's responses. These plans were evaluated against a reference management plan based on American Diabetes Society guidelines. Completeness, necessity, and dosage accuracy were quantified and a Prescribing Error Score was devised to assess the quality of the generated management plans. The safety of the management plans generated by GPT-4 was also assessed.

Results

Results indicated that physicians' management plans had fewer missing medications compared to those generated by GPT-4 (p=0.008). However, GPT-4-generated management plans included fewer unnecessary medications (p=0.003). No significant difference was observed in the accuracy of drug dosages (p=0.975). The overall error scores were comparable between physicians and GPT-4 (p=0.301). Safety issues were noted in 16% of the plans generated by GPT-4, highlighting potential risks associated with AI-generated management plans.

Conclusion

The study demonstrates that while GPT-4 can effectively reduce unnecessary drug prescriptions, it does not yet match the performance of physicians in terms of plan completeness. The findings support the use of LLMs as supplementary tools in healthcare, highlighting the need for enhanced algorithms and continuous human oversight to ensure the efficacy and safety of artificial intelligence in clinical settings.

## Introduction

As the frontier of artificial intelligence (AI) continues to advance, the integration of large language models (LLMs) such as GPT-4 into healthcare settings presents both promising opportunities and significant challenges. The potential of LLMs to enhance healthcare education, research, and practice is noteworthy, with applications ranging from improving scientific writing to assisting in complex data analysis and personalized medicine. However, the deployment of these technologies in clinical environments must be approached with caution due to concerns about accuracy, ethical considerations, and the potential for bias [1].

LLMs like GPT-4 [2] have shown significant potential in a range of healthcare applications, from generating patient management plans to assisting with medical documentation. Despite their capabilities, the accuracy and utility of these models in practical, clinical settings require thorough evaluation and benchmarking against established medical standards. A recent systematic review has noted that while LLMs perform well in tasks like answering medical exam questions, their application in direct patient care and other complex medical scenarios remains underexplored and often lacks integration with real patient data [3].

Moreover, the evaluation of these models in healthcare has often focused narrowly on specific tasks, such as NLP tasks related to summarization and conversation, without a broad application across various medical specialties. This has limited the understanding of their broader potential and areas where they may not perform as expected. To truly harness the capabilities of LLMs like GPT-4, comprehensive assessments using real-world data and across diverse healthcare tasks are essential [1].

The advanced capabilities of LLMs may extend beyond routine natural language processing to include complex clinical interactions and decision-making processes. It is necessary to carry out a more accurate assessment of LLMs' functionality in real-world clinical scenarios [4].

A few recent studies have been conducted to evaluate LLMs in a clinical context. A study assessed GPT-4's orthopedic treatment recommendations using MRI reports. Findings showed generally accurate advice but noted limitations in context awareness and specificity, emphasizing the need for professional oversight [5]. Another study compared GPT-3.5, GPT-4, and other LLMs with ophthalmologists in mock exams. GPT-4 outperformed other models and approached expert-level accuracy, suggesting potential for clinical application [6]. Another study evaluated five LLMs on oncology questions, demonstrating heterogeneity in performance. GPT-4 excelled, outperforming other models and showing potential for clinical oncology application, though all models displayed significant error rates [7].

A recent systematic review highlights the expanding role of LLMs, including GPT-4, in health care. While most evaluations focus on medical knowledge assessments and diagnostic tasks, research on their application in real-world patient care remains limited. The review underscores the need for standardized evaluation frameworks that address fairness, bias, and deployment challenges. Further studies are required to assess LLMs’ potential in tasks such as personalized treatment recommendations and clinical decision support [3].

Research involving a comparative analysis of hypothetical diabetes cases assessed by GPT-4 against expert evaluations indicates a high concordance, suggesting LLMs' capability to support healthcare professionals in diabetes management. However, discrepancies in complex clinical judgments call for further refinement of AI technologies [8].

This study aims to fill these gaps by directly comparing the management plans created by GPT-4 with those devised by physicians, focusing on type 2 diabetes, a prevalent and complex medical condition. The comparison will consider several dimensions of evaluation, including the accuracy, comprehensiveness, and practical usability of the management plans, providing a clearer picture of where LLMs stand in terms of replacing or augmenting traditional healthcare processes.

By addressing these critical points, the article will contribute valuable insights into the current capabilities and future potential of LLMs in healthcare, informing both technological developers and healthcare professionals about the strengths and limitations of these advanced AI tools in managing chronic diseases like type 2 diabetes.

This article was previously posted to the medRxiv preprint server on May 22, 2024 [8].

## Materials and methods

This comparative study evaluated the performance of GPT-4, against management plans created by physicians for type 2 diabetes. The study was designed to compare key metrics in three domains including completeness, necessity, and accuracy.

The objectives of our study were as follows: (1) to evaluate the performance of GPT-4 in generating management plans for type 2 diabetes mellitus compared to physicians; and (2) to analyze the safety of AI-generated management plans, identifying potential risks and limitations.

We hypothesized that GPT-4 would generate management plans for type 2 diabetes mellitus that are comparable to those created by physicians. While AI has the potential to assist in clinical decision-making, we expected its performance to align closely with that of physicians, demonstrating similar effectiveness in treatment recommendations while requiring human oversight to ensure patient safety.

The study involved anonymized records of patients with type 2 diabetes mellitus from a healthcare database from the private practice of the second author in West Bengal, India.

The study included records of patients of all ages and sexes with type 2 diabetes mellitus from the past year. These records contained comprehensive patient data, including case summaries, laboratory reports, and medication history. Incomplete records with missing clinical or laboratory data were excluded.

Management plans for each of the selected patient records were generated through both GPT-4 and the physicians. Three physicians created the management plans independently who were blinded to the responses generated by each other. The application programming interface (API) of GPT-4 was used for generating the management plans.

A reference management plan was carefully developed in collaboration with the first two authors using the American Diabetes Society (ADA) guidelines [9]. The management plans generated by the physicians and GPT-4 were evaluated against this reference.

To assess the reliability of ratings among three physicians, we computed the Intraclass Correlation Coefficient (ICC) using a two-way random effects model with consistency and average measures, accompanied by an F-test for statistical significance.

As there was no preexisting metric suitable for such a comparative study between large language model and physicians, we devised a Prescribing Error Score for this purpose as follows:

$ES = \frac{MD + UD + ID}{N + 1}$

Where: ES = Error Score; MD = number of missing drugs in the management plan; UD = number of unnecessary drugs included in the management plan; ID = number of inaccurate dosages among the correctly included drugs; N = number of drugs the patient was already on during evaluation.

We calculated the error score to account for all three domains including completeness, necessity, and dosage accuracy. We also included the number of drugs the patient was already on to account for the potential complexity of the case and we added one to it to avoid division by zero if a patient presents for the first time with no prior medication.

The number of missing drugs, the number of unnecessary drugs, and the number of inaccurate doses were determined based on the comparison with the reference management plan. The error score was calculated for each individual management plan.

**Figure 1:**
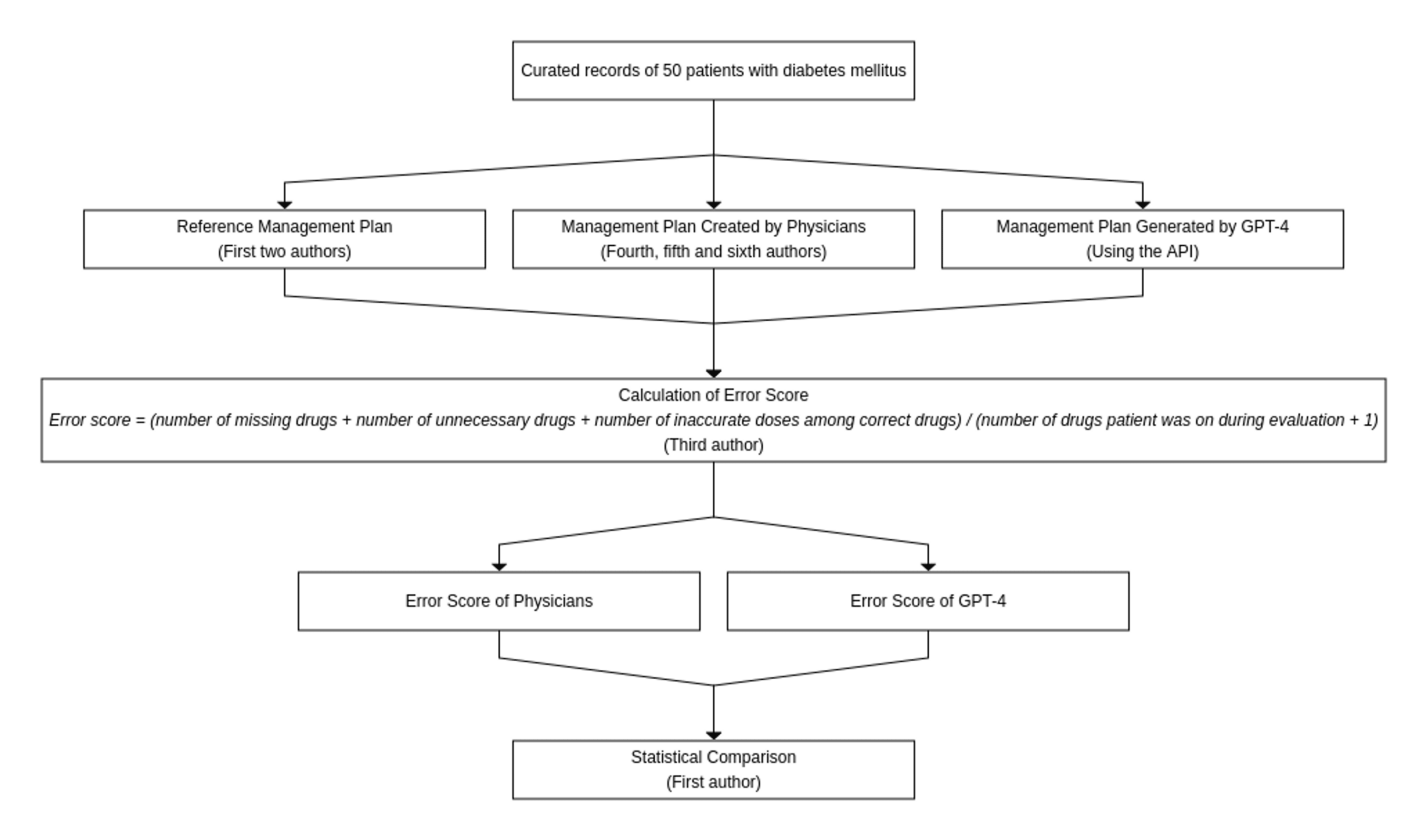
Methodological Framework for Comparing Physician and AI-Generated Diabetes Management Plans

Management plans generated by each physician as well as the one generated by GPT-4 were evaluated separately against the reference by the third author who did not participate in the preparation of the reference management plans. Each of the 50 management plans was scored separately for each physician and GPT-4. The error scores of the three physicians were then averaged for each management plan and the average error scores were used for comparison with the error scores obtained by GPT-4. The methodology is shown as a flowchart in Figure 1.

In addition, the management plans generated by GPT-4 were separately evaluated for potential safety issues. However, it was not included in the comparison.

Statistical analysis was conducted using R software (R Foundation for Statistical Computing, Vienna, Austria). The primary analysis involved a comparison of average error scores obtained by the physicians and those obtained by GPT-4. The error scores were described with mean and standard deviation (SD). Wilcoxon rank-sum test was used for the comparison. The effect size was shown with a 95% confidence interval (95% CI). A p-value of less than 0.05 was considered significant.

The study protocol was reviewed and approved by Clinimed Independent Ethics Committee, Kolkata on May 11, 2024, with the reference number CLPL/CIEC/001/2024. All case records were deanonymized prior to inclusion in the study and data handling procedures strictly followed ethical guidelines.

The study was conducted over a total duration of four weeks with data collection and analysis being completed over a period of one week following the approval by the ethics committee.

## Results

This study compared the management plans for the records of 50 patients with type 2 diabetes mellitus generated by physicians and GPT-4 against a reference plan in accordance with the ADA guidelines.

Inter-rater reliability among physicians showed moderate agreement across the three assessment areas. The ICC values were 0.672 (95% CI: 0.475 to 0.803, p < 0.001) for completeness, 0.512 (95% CI: 0.22 to 0.707, p = 0.001) for necessity, and 0.588 (95% CI: 0.341 to 0.752, p < 0.001) for dose accuracy.

Completeness

The mean number of missing drugs in the management plan generated by the physicians was 1.15 (SD 0.8). In comparison, the mean number of missing drugs in the GPT-4 generated management plan was 1.76 (SD 1.2). The difference between these two was significant (p=0.008).

Necessity

The mean number of unnecessary drugs in the management plan created by the physicians was 0.68 (SD 0.64) while it was 0.4 (SD 0.61) in the management plan generated by GPT-4. The difference was significant (p=0.003).

Dose accuracy

The mean number of inaccurate dosages of correctly included drugs was 0.74 (SD 0.58) in management plans generated by the physicians and 0.86 (SD 0.9) in those by GPT-4. The difference was not significant (p=0.975).

Error score

**Figure 2:**
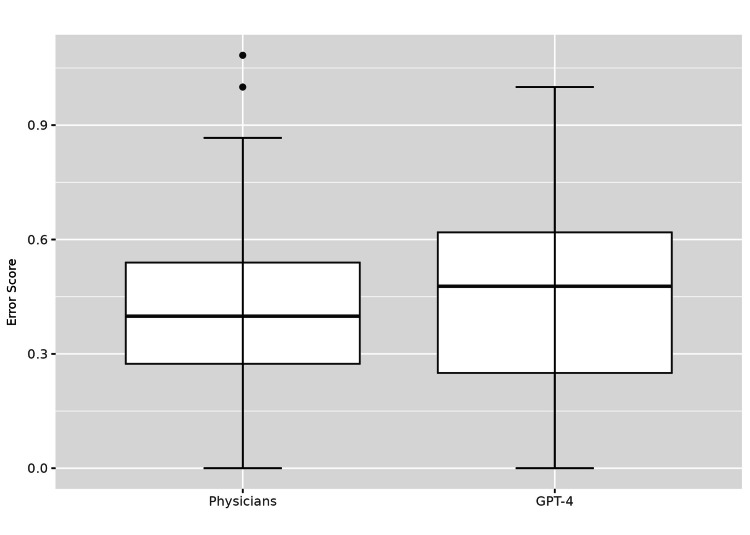
Comparison of Error Scores: Physicians vs. GPT-4 in Type 2 Diabetes Management

The mean error score of the management plans formulated by the physicians was 0.41 (SD 0.23) while it was 0.46 (SD 0.25) for GPT-4. A boxplot showing the comparison is shown in Figure 2. There was no significant difference between the physicians and GPT-4 in this regard (p=0.301). The difference in location was -0.05 (95% CI -0.14 to 0.05). The findings of the study are shown in Table 1.

**Table 1:** Comparative Analysis of Physicians and GPT-4 in Type 2 Diabetes Management Values are presented as mean ± standard deviation. Statistical comparisons were performed using the Wilcoxon rank sum test. A p-value of less than 0.05 was considered statistically significant. * Averaged score of three physicians. † Mean ± standard deviation

Variables	Physicians* ^†^	GPT-4 ^†^	p-value	Difference in location	95% Confidence interval
Number of missing drugs	1.15 ± 0.8	1.76 ± 1.2	0.008	-0.67	-1.0 to -0.33
Number of unnecessary drugs included	0.68 ± 0.64	0.4 ± 0.61	0.003	0.33	0 to 0.67
Number of inaccurate doses among the correctly included drugs	0.74 ± 0.58	0.86 ± 0.9	0.975	0	-0.33 to 0.33
Error score	0.41 ± 0.23	0.46 ± 0.25	0.301	-0.05	-0.14 to 0.05

Safety

There were safety issues in eight (16%) management plans generated by GPT-4. Five of them were due to an elevated risk of hypoglycemia and three were due to safety concerns with the drugs used. In one case GPT-4 did not withhold SGLT2 inhibitor despite recurrent urinary tract infection. In another case, GPT-4 continued saxagliptin despite congestive heart failure. In another case, GPT-4 increased the dose of atorvastatin despite raised liver enzymes.

Referral

In the management plans generated by GPT-4, the referrals were generally appropriate and comprehensive including referrals to ophthalmologists, nephrologists, and other specialties. In only one case (2%) GPT-4 failed to refer the case to a nephrologist despite deteriorating renal function.

## Discussion

This study provides important insights into the use of LLMs, particularly GPT-4, in generating management plans for type 2 diabetes mellitus compared to those formulated by physicians. Our analysis assessed four critical aspects: completeness, necessity, dose accuracy, and safety of the management plans.

Our findings indicate that physicians created more complete management plans than GPT-4, with fewer missing drugs on average (1.15 vs. 1.76, p=0.008). While GPT-4 demonstrates the ability to generate management plans, it occasionally omits necessary medications, potentially reducing treatment effectiveness. The higher standard deviation in GPT-4-generated plans suggests variability in completeness, which could be addressed through enhanced training algorithms to ensure consistency in necessary medication inclusion.

On the other hand, GPT-4 included fewer unnecessary medications compared to physicians (0.4 vs. 0.68, p=0.003), highlighting its strength in adhering to essential treatment protocols. This advantage likely stems from its capacity to leverage extensive datasets, reducing prescriber bias and variability in medication selection.

Regarding dosage accuracy, there was no significant difference between the groups (p=0.975). Both physicians and GPT-4 displayed similar capabilities in dosing accuracy, reinforcing the potential of AI as a supportive tool for managing complex conditions like diabetes, where precision is essential. The overall error scores between the two groups were also statistically insignificant (p=0.301), suggesting that GPT-4 can produce management plans of a quality comparable to those of medical professionals.

We also assessed inter-rater reliability among physicians using the ICC, which indicated moderate reliability, suggesting a consistent level of agreement in assessments, though some variability persists. This reinforces the validity of physician-generated management plans while acknowledging inherent differences in clinical decision-making.

However, a significant concern arising from our study is the safety of AI-generated plans. Safety issues were identified in 16% of GPT-4-generated plans, with risks including hypoglycemia and inappropriate medication continuation despite contraindications such as recurrent urinary tract infections, congestive heart failure, and elevated liver enzymes. These findings emphasize the need for AI models to integrate complex clinical scenarios and patient histories more effectively, underscoring the necessity of human oversight to prevent potential safety hazards.

GPT-4 demonstrated high accuracy in generating appropriate and comprehensive referrals, showcasing its potential to assist healthcare professionals in streamlining patient management and facilitating timely specialist consultations. However, occasional oversights, such as missed nephrology referrals, highlight the need for continued monitoring and validation of AI-generated recommendations.

In considering technological innovations in healthcare, it is evident that while AI interventions often perform comparably to traditional methods in controlled settings, real-world performance may vary [10]. To assess GPT-4’s practical applicability, we compared its outputs with those of physicians using real patient records.

Recent advancements in diabetes management have underscored the importance of not only controlling blood sugar levels but also preventing complications and managing the disease’s systemic effects [11]. For LLMs to be effectively integrated into clinical care, they must go beyond replicating guideline-based recommendations and provide nuanced, patient-specific decision support.

Despite significant research and the implementation of new treatment protocols, such as the increased use of GLP-1 receptor agonists and SGLT2 inhibitors, managing type 2 diabetes remains a complex challenge requiring a multifaceted approach [11]. Automated systems like GPT-4, while capable within the scope of their training, do not yet surpass human expertise in clinical decision-making and personalized patient care.

Although AI can enhance pharmacotherapy in chronic disease management, clinician oversight remains essential for adherence, lifestyle interventions, and shared decision-making. [10]. While LLMs can enhance efficiency by providing data-driven insights and reducing administrative burdens, human judgment remains indispensable in managing chronic diseases like type 2 diabetes [12].

Our findings align with the study by Flory et al., which compared GPT-4’s decision-making with that of endocrinologists and found that the model’s treatment choices often diverge from expert clinical judgment, particularly in cases of clinical uncertainty. A notable similarity is GPT-4’s conservative prescribing tendencies, as seen in its lower selection rate of metformin in scenarios involving kidney impairment or gastrointestinal distress [13]. However, while our study demonstrated GPT-4’s ability to reduce unnecessary prescriptions, it also identified safety concerns in 16% of generated management plans, reinforcing the need for rigorous oversight. These findings collectively emphasize that while LLMs have the potential to function as valuable supplementary tools, they should not replace clinical expertise until they can consistently ensure safety, completeness, and adherence to both guidelines and real-world clinical decision-making.

Overall, our study highlights the potential of LLMs as supportive tools rather than replacements in healthcare. Further research and development are necessary to refine their application in clinical settings. Future studies should explore how AI can be optimally integrated into diabetes care, particularly in managing complex cases that require a balance of medication, lifestyle interventions, and patient education.

This study has several limitations that should be considered when interpreting the results. Our sample size of 50 patient records is relatively small and may not capture the full spectrum of variability in type 2 diabetes management across different populations and healthcare environments. Additionally, although the evaluation of management plans against ADA guidelines was conducted by physicians, the potential for subjective bias in assessment remains. To mitigate this bias, we developed a reference management plan for comparison. Future research should explore standardized, automated scoring systems to enhance objectivity and reproducibility in evaluating AI-generated plans.

AI technologies, including LLMs, are rapidly evolving. The findings of this study may not remain applicable as newer versions and alternative AI models emerge, potentially improving effectiveness and safety in clinical applications. Future research should focus on several key areas to advance the application of AI in managing type 2 diabetes and other chronic conditions. Studies should incorporate a larger and more diverse dataset from multiple healthcare settings worldwide to enhance generalizability and evaluate AI performance across various clinical contexts.

The evolving nature of AI models necessitates consideration of the study’s timing, as newer iterations may yield different results. Future studies conducted at different time points could provide valuable insights into the progression and refinement of AI-driven clinical decision-making. An important consideration for future research is the potential presence of systematic biases in GPT-4’s recommendations, such as tendencies in medication selection. Evaluating these patterns could provide deeper insights into AI-driven prescribing behaviors and their implications in clinical practice.

Exploring the impact of different training datasets on AI performance is crucial. Future studies should investigate AI training with a broad range of patient scenarios, including those with multiple comorbidities, to assess how well AI adapts to complex clinical situations. Additionally, the role of real-time learning and continuous adaptation in AI systems should be examined to determine their potential to evolve with ongoing use in clinical practice.

Developing automated systems for evaluating AI-generated management plans against clinical guidelines could reduce human bias and improve reproducibility. Future research should focus on creating and validating such systems, which could also serve as real-time clinical decision-support tools. Longitudinal studies tracking patient outcomes over time will provide critical insights into the long-term effectiveness and safety of AI-supported treatment plans. Furthermore, pilot studies testing AI systems in real-world clinical settings could assess practical challenges and their impact on patient outcomes.

As AI becomes more integrated into healthcare, it is essential to explore ethical considerations and establish robust regulatory frameworks to ensure patient safety and data privacy. Additionally, studies should examine healthcare providers’ and patients’ perceptions of AI to identify and address potential barriers to adoption. Comparative studies assessing different AI models, including newer versions of GPT and other platforms, will help determine which models are most effective for specific aspects of diabetes management. This research will guide healthcare professionals in selecting the most suitable AI tools for their needs.

## Conclusions

This study provides valuable insights into the capabilities and limitations of LLMs in managing type 2 diabetes mellitus. While AI demonstrates potential in reducing unnecessary prescriptions, it falls short in completeness and safety compared to physicians. Although GPT-4 matches human performance in some aspects, such as dosage accuracy and overall error scores, significant concerns remain regarding its ability to handle complex patient conditions safely. These findings underscore the continued need for human oversight in reviewing AI-generated treatment plans, particularly in nuanced clinical scenarios.

## Appendices

Table 2 demonstrates the GPT-4 API configuration used to generate diabetes management plans.

**Table 2:** GPT-4 API Configuration for Generating Diabetes Management Plans The input to GPT-4 consisted of patient history, laboratory reports, and existing medications, all provided as structured text. The AI-generated management plan was based solely on this textual information. To maintain consistency, only the first response from GPT-4 was used, as the temperature was set to 0, ensuring deterministic outputs. This approach eliminated variability in responses and simulated a real-world scenario where a single AI-generated recommendation is reviewed by healthcare professionals.

Model	gpt-4
Temperature	0
System prompt	You are a healthcare professional, specialized in diabetes care. Follow ADA guideline for diabetes management. You will review the case histories provided to you and generate a management plan. Be brief and only mention the management plan in bullet points. Clearly separate the drug changes and other advice. Do not use markdown formatting. Write referrals to other specialties as necessary.
